# Chest Wall Motion Model of Cardiac Activity for Radar-Based Vital-Sign-Detection System

**DOI:** 10.3390/s24072058

**Published:** 2024-03-23

**Authors:** Shaocan Fan, Zhenmiao Deng

**Affiliations:** School of Electronics and Communication Engineering, Shenzhen Campus of Sun Yat-sen University, Shenzhen 518107, China; fanshc3@mail2.sysu.edu.cn

**Keywords:** CWM model, heartbeat, data, non-contact monitoring, Doppler radar

## Abstract

An increasing number of studies on non-contact vital sign detection using radar are now beginning to turn to data-driven neural network approaches rather than traditional signal-processing methods. However, there are few radar datasets available for deep learning due to the difficulty of acquiring and labeling the data, which require specialized equipment and physician collaboration. This paper presents a new model of heartbeat-induced chest wall motion (CWM) with the goal of generating a large amount of simulation data to support deep learning methods. An in-depth analysis of published CWM data collected by the VICON Infrared (IR) motion capture system and continuous wave (CW) radar system during respiratory hold was used to summarize the motion characteristics of each stage within a cardiac cycle. In combination with the physiological properties of the heartbeat, appropriate mathematical functions were selected to describe these movement properties. The model produced simulation data that closely matched the measured data as evaluated by dynamic time warping (DTW) and the root-mean-squared error (RMSE). By adjusting the model parameters, the heartbeat signals of different individuals were simulated. This will accelerate the application of data-driven deep learning methods in radar-based non-contact vital sign detection research and further advance the field.

## 1. Introduction

Non-contact radar vital sign detection has become a popular research topic in recent years [1,2,3,4,5]. Most studies have focused on extracting vital sign information from radar signals, particularly the estimation of the respiration rate and heart rate [6]. Estimating the respiration rate is usually relatively straightforward since the displacement of the chest cavity during breathing is significantly larger than that caused by the heartbeat. Detecting weak heartbeat signals has been a major challenge in the field of non-contact radar vital sign detection [7]. Research has demonstrated that heartbeat signals collected using radar (Doppler cardiogram (DCG)) under breath-hold conditions exhibit a high degree of correlation with electrocardiogram (ECG) data [3,8,9]. The heart rate variability (HRV) obtained from DCG provides information about heart-related diseases and is also a key indicator of an individual’s health status [10,11]. However, this conclusion assumes that the heartbeat signal can be extracted accurately from the mixed signal. Unfortunately, detecting heartbeat signals can be complex due to interference from respiratory signals [12,13].

Due to the limitations of the radar beam width, it is impossible to focus the beam exclusively on the heart or to ensure that only the area containing the respiratory signal is irradiated. Consequently, in practical applications, respiratory and heartbeat signals often intertwine and are challenging to separate completely. Conventional signal-processing methods, like the use of band-pass filters, can filter out some noise. However, they might also distort the waveform and lose its physical significance. The decomposition–reconstruction method aims to decompose a complex signal into multiple components. The desired target signal components are then selected for reconstruction. Two commonly used decomposition methods are wavelet decomposition (WTD) [14] and empirical-mode decomposition (EMD) [15]. However, WTD faces the problem of respiratory harmonic aliasing when processing the signal. This issue can lead to the reconstructed signal containing other frequency components, making it difficult to obtain a pure heartbeat signal. In contrast, EMD is a data-driven decomposition method that can decompose non-smooth signals into different intrinsic mode functions (IMFs). Although EMD is not affected by the choice of the wavelet basis function or decomposition level, it is difficult to determine which signal component represents the heartbeat waveform through EMD decomposition without considering mode mixing and end effects [16]. As a result, the reconstructed signal may suffer from waveform distortion. Due to the difficulty in obtaining accurate heartbeat waveforms using the aforementioned methods, numerous researchers resort to utilizing neural networks [17,18,19,20]. In our previous work, we utilized neural networks to estimate and recover heartbeat signals from mixed signals. We introduced the end-to-end TFA-Net model for the time–frequency analysis of signals [21]. However, the model did not yield the expected results in processing the mixed signals of respiration and heartbeat captured by the radar. This was due to the use of sinusoidal signals with Gaussian noise added to the training data. Therefore, a high-quality dataset is crucial to accurately estimate and recover heartbeat signals from mixed signals using neural networks. Unfortunately, there are only a few publicly available datasets related to radar vital sign detection. Additionally, none of the existing datasets provide separate heartbeat signals, such as seismocardiography (SCG) acquired by an accelerometer, during normal breathing. This further demonstrates the urgency and importance of building an accurate model of heartbeat-induced chest wall displacement.

Using a sinusoidal waveform as a model [22] to simulate heartbeat signals is the simplest approach. However, it is crucial to note that the heartbeat signal is, in fact, a pseudo-periodic signal that does not strictly adhere to a sinusoidal motion. Furthermore, the heartbeat exhibits distinct characteristics at different stages and is not a smooth signal. Curve fitting enables the direct modeling of signals. While a perfect fit to the DCG can be achieved, it is not recommended as it may lead to overfitting. Additionally, generating waveforms with different features can be difficult once the model parameters are set. This limitation hinders the model’s potential to generate diverse data for use in scenarios like neural network training. Therefore, basic curve-fitting techniques are inadequate when generating waveforms with supplementary characteristics for training or testing purposes.

In [23], the authors proposed that rhythmic electrical stimuli produced by the sinus node lead to corresponding rhythmic behavior in the myocardium. Since their rhythms are similar, the van der Pol equation was used to describe this resonant motion. However, modeling the complex process of electrical stimulation, myocardial response, and myocardium-induced cardiac contraction and diastole on the chest wall is problematic. Directly using the rhythmic behavior of the sinus node is also a challenging. The solution to the van der Pol equation heavily depends on the given parameters, and the limited number of adjustable parameters hinders effective control over the period and frequency of the generated waveform. Mehrdad et al. proposed a Gaussian pulse train model for simulating heartbeat signals for HRV analysis [24]. The model imitates the behavioral characteristics of photoplethysmography signals. By defining the interval between each pulse, a heartbeat waveform with HRV characteristics can be generated. However, this waveform differs significantly from the chest wall motion (CWM) waveform acquired by actual radar. SCG is a technique that measures the acceleration of the chest wall caused by myocardial motion. By quadratic integration of the SCG signal, the displacement of the chest wall can be derived. Mehrdad et al. proposed an improved Gaussian pulse waveform model based on this principle [25]. However, there has been no comprehensive study in the literature on the correlation and similarity between the SCG obtained through acceleration and velocity signals obtained by radar. Compared to the previous model, the waveform generated by the new model has the same drawbacks.

The aim of this study is to accurately model the CWM. The contributions of this paper are as follows:

- We demonstrate consistent patterns of the CWM induced by the heartbeats using two different acquisition systems: the VICON Infrared (IR) motion capture system and continuous wave (CW) radar system.
- Identified and generalized CWM characteristics specific to each phase of the cardiac cycle.
- By combining physiological and CWM characteristics, we constructed a mathematical model of the CWM. We verified the model’s ability to generate highly realistic heartbeat-related CWM data through qualitative analysis, quantitative analysis, and spectral analysis. This outcome provides accurate and high-quality simulation data to support data-driven deep learning.

The paper is structured as follows: Section 2 provides an in-depth analysis of the publicly available VICON and 24 GHz CW radar data. The aim is to summarize the pattern of heartbeat-induced CWM during breath-holding. A mathematical model will be constructed to describe the CWM based on the previously summarized pattern and physiological features. In Section 3, we will analyze the model-generated data both qualitatively and quantitatively and compare it with the actual collected data to verify the model’s rationality and validity. Section 4 discusses the limitations and potential applications of the model. Finally, Section 5 provides the conclusion.

## 2. Materials and Methods

### 2.1. CWM Analysis Based on Camera and Continuous Wave Radar

#### 2.1.1. Introduction to the Datasets

Shafiq et al. [26] published a multimodal chest surface motion dataset for respiratory and cardiovascular monitoring. The dataset includes surface motion signals captured on the chest surface using the VICON IR motion capture system, nasal breathing signals captured using a thermal sensor, chest expansion signals captured using a strain belt during respiration, and an electrocardiogram in lead-II configuration. All data were synchronously acquired. The dataset includes data from 11 participants across four conditions: normal breathing, breath-hold, irregular breathing, and post-exercise recovery. The study demonstrated the similarity between heartbeat intervals and R–R intervals obtained from chest motion (the z-axis of marker L22 in the VICON system under breath-holding) and ECG.

We selected four recordings with stable waveforms. Four data were selected from which the waveforms were stable. Schellenberger et al. published a dataset of clinically recorded radar vital signs and synchronized reference sensor signals [3]. The data included the electrocardiogram, impedance cardiogram, and non-invasive continuous blood pressure, which were synchronized with a 24 GHz frequency modulation continuous-wave radar based on Six-Port technology. The study involved thirty subjects in different scenarios, such as resting, Valsalva, and apnea tilt up and tilt down. For the analysis, we selected the radar and synchronized ECG data from the apnea scenario. From these, 13 data with stable waveforms were selected. It should be noted that the data for both datasets were collected from healthy individuals. For all data used for the study, the ratio of males to females was 11:6 and the age was 30.9±6.7. The detailed information of all the subjects is listed in Table 1.

#### 2.1.2. Synchronized ECG Signal Feature Point Auto-Calibration

The Pan–Tompkins algorithm [27,28] was employed for detecting the R peaks in the ECG signal. Subsequently, the P, Q, R, S, and T waves were automatically delineated based on the temporal intervals and positional relationships inherent in the PQRST complex. To ensure the precision of these feature points, all automated delineations were meticulously reviewed and manually corrected as necessary.

#### 2.1.3. CWM Velocity Analysis

**Figure 1:**
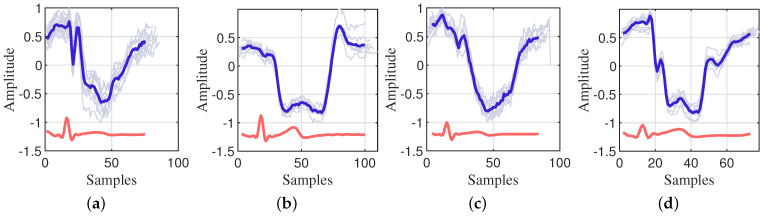
The CWM captured by the VICON system. (**a**–**d**) are the CWM from 4 participants. 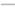 each radar cardiac cycle according to the ECG cardiac cycle; 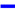 average of all radar cardiac cycles; 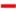 average of all ECG cardiac cycles.

**Figure 2:**
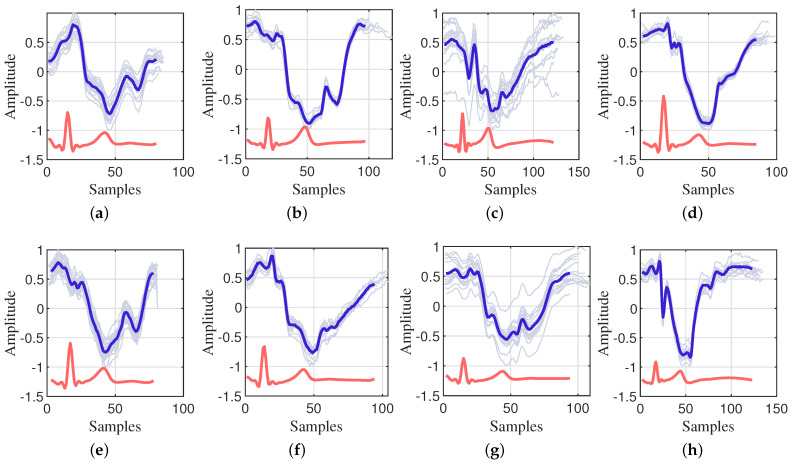
The CWM captured by 24 GHz CW radar. (**a**–**h**) are the CWM from 8 participants. 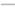 each radar cardiac cycle according to ECG cardiac cycle; 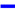 average of all radar cardiac cycles; 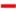 average of all ECG cardiac cycles.

We conducted an analysis of chest wall heartbeat displacements utilizing data captured by the VICON system and a 24 GHz CW radar during breath-holding. The segmentation of chest wall displacement periods and the characterization of each phase were derived from electrocardiogram (ECG) feature points. Specifically, we employed the current position of the P-wave (atrial depolarization wave) as the starting point and the subsequent cycle’s P-wave position as the endpoint to delineate each cardiac cycle. The signals from each cycle were normalized to ensure the comparability and analyzability of the data. It is essential to acknowledge that, due to the pseudo-periodicity of the heartbeat signal, there exists a certain degree of error in the time intervals between heartbeats. To ensure precise alignment of each cycle, we selected the R-wave (ventricular depolarization wave) as the reference point. Figure 1 and Figure 2 present a detailed representation of the CWM signals, the ECG signals, and their respective mean values for each cycle from two datasets. It is evident that distinct cardiac cycles not only vary in cycle length, but also exhibit slight differences in details, such as changes in amplitude and trend.

**Figure 3:**
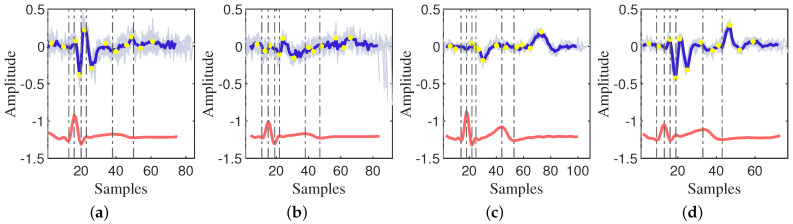
The velocity of the CWM captured by the VICON system. (**a**–**d**) are the CWM from 4 participants. 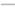, velocity of each radar cardiac cycle according to the ECG cardiac cycle; 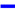, average of all radar cardiac cycles; 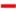, average of all ECG cardiac cycles; 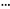, characteristic points of the ECG; 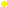, characteristic points of the velocity of the radar cardiac cycle.

**Figure 4:**
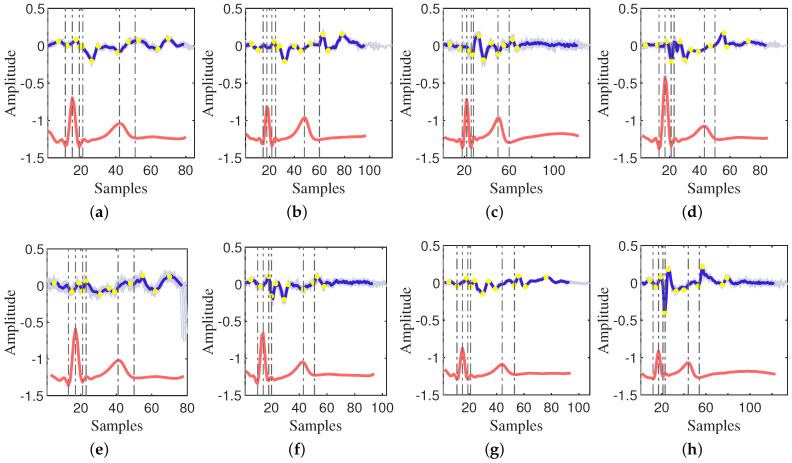
The velocity of the CWM captured by the 24 GHz CW radar. (**a**–**h**) are the CWM from 8 participants. 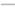, velocity of each radar cardiac cycle according to ECG cardiac cycle; 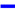, average of all radar cardiac cycles; 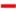, average of all ECG cardiac cycles; 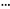, characteristic points of the ECG; 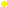, characteristic points of the velocity of the radar cardiac cycle.

The velocity variation curve, derived from the amplitude variation curve, is illustrated in Figure 3 and Figure 4. It is apparent from the figures that there is a notable jitter in velocity between two points due to the large sampling interval, resulting in a visually discontinuous curve.

Utilizing characteristic points, namely P, Q, R, S, and T, calibrated in the ECG, the phases of the cardiac cycle are accurately delineated. This classification enables the categorization of the CWM into the isovolumetric systole, ejection phase, isovolumetric diastole, filling phase, and atrial systole phase. The examination of the velocity change characteristics within each phase forms a robust foundation for subsequent modeling.

Starting with the atrial systole phase, the observed patterns in Figure 3 and Figure 4 are as follows:

- After the peak of the P-wave, the velocity initially increases from 0 and subsequently decreases. During this period, there is an extreme positive value followed by a negative extreme value near the Q-wave.
- A peak is observed within the RS segment (isovolumetric contraction (IVC)) of the ECG.
- There may be a localized minimum near the J-point of the ECG.
- The $J-T_{end}$ segment exhibits two local minima and two local maxima. The two minima occur near the beginning and peak of the T-wave, respectively.
- Near the end of the T-wave, the end-systolic velocity reduces to zero.
- Isovolumetric relaxation (IVR) velocities exhibit an initial increase, followed by a decrease, potentially reaching negative minima at the end of IVR.
- Velocity undergoes rapid transition from negative (or 0) into the rapid-filling (RPF) phase, featuring a positive localized extreme value, followed by a gradual decrease.

In the modeling process, the physiological properties of the cardiac cycle will be combined, and the previously described rules will be followed to construct the CWM model. Based on the characteristic points of the motion velocity of the CWM as depicted in Figure 3 and Figure 4, a cardiac cycle will be divided into 10 phases. The first task is to determine the duration of each phase in the cardiac cycle to ensure that the model accurately reflects the real physiological situation. Subsequently, appropriate functions will be selected for fitting based on the characteristics of the velocity change waveforms of each phase in the actual signal. The choice of function is crucial to the accuracy and practicality of the model. When selecting the function, the shape, trend, and characteristics of the velocity change curve, as well as the transition and interaction between the stages will be considered.

### 2.2. The Proposed Heartbeat Model

#### 2.2.1. Determination of the Duration of Each Phase of the Cardiac Cycle

First, the duration of a cardiac cycle, denoted as cycle_len, is determined. The resting heart rate of a normal subject is in the range of 60 to 100 beats per minute (bpm) [29]. To account for more extreme cases, a range between 56 and 102 bpm is established, resulting in cycle_len ranging from 0.58 s to 1.07 s. A Gaussian distribution with a mean of 0.82 and a standard deviation of 0.08 is used to generate a sequence of heartbeat cycle duration data. In general, systole tends to be shorter than diastole. The systolic and diastolic durations are represented by $L_{sys}$ and $L_{dia}$. The ratio of systole to diastole, denoted as *r*, is set in the range [0.7, 0.9] using a uniform distribution. Once cycle_len is determined, the systolic and diastolic durations can be calculated. The ratio of the IVC to the systole, denoted as $r_{ivc}$, is established between 0.09 and 0.13. It is generated via a uniform distribution. The duration of IVC is represented as $L_{ivc}$. The occurrence of IVC is during the RS intervals within the QRS complex. The duration of the QRS complex, denoted as $L_{qrs}$, is established within the range of $\left[1.8,2.5\right]\times L_{ivc}$. To ensure that the maximum duration of the QRS complex does not exceed 120 ms, constraints are implemented on $L_{qrs}$.

In the absence of a clear demarcation between the rapid ejection periods (RPEs) and reduced ejection periods, the modeling approach, which is based on the measured data, no longer divides the ejection period into these two phases. An analysis of the signals from both the camera and radar indicates that the changes in velocity during the ejection period are rapid and complex. As a result, a more granular temporal division during the ejection period has been justified. Upon referring to Figure 3 and Figure 4, it becomes evident that identifying the locations of the S-wave, J-wave, the onset of the T-wave, and the peak of the T-wave are crucial. The J-point, which is the junction where the QRS complex meets the ST segment, signifies the approximate end of depolarization and the beginning of repolarization. However, the specific time interval between the S-wave and the J-point is not usually measured nor reported in standard ECG interpretation. This time duration, represented as $L_{sj}$, was analyzed from the available camera and radar data and was found to be within the range [0.01,0.03]. By employing a uniform distribution, $L_{sj}$ is randomly generated within this range.

The positions of the T-wave start and T-wave peak are denoted by $T_{start}$ and $T_{peak}$, respectively. The ST segment, which connects the QRS complex and the T-wave, begins at the J-point and ends at the initiation of the T-wave. The typical duration of the ST segment is around 0.08 s (80 ms). To account for individual differences, the duration of the ST segment, represented as $L_{st}$, is randomly generated between 70 ms and 90 ms using a uniform distribution.

The duration of the isovolumetric relaxation (IVR) phase is proportionally generated, with its proportion to the diastolic period falling within the range [0.09,0.13]. This generation is executed through a uniform distribution. In the absence of a clear demarcation between the RPF periods and reduced-filling periods (RDFs), the duration for the RPF phase is set between [0.25,0.4], denoted as $L_{rpf}$, and is generated using a uniform distribution. The proportion of the atrial systole phase to the diastolic phase is established within the range of 16% to 22%, denoted as $L_{asys}$, and is generated using a uniform distribution. Simultaneously, it is ensured that the duration of the atrial systole significantly exceeds that of the QRS complex. The total duration of the diastole, which comprises the IVR phase, RPF phase, reduced-filling phase, and atrial systole phase, is calculated. With the durations of the other three phases known, the duration of the reduced-filling phase, denoted as $L_{rdf}$, can be calculated using the formula $L_{rdf}=L_{dia}-L_{rpf}-L_{asys}$. Finally, a physiologically realistic distribution of the duration for each phase of the cardiac cycle is generated using either a Gaussian distribution or a uniform distribution.

#### 2.2.2. Fitting the Velocity Using a Piecewise Function

Atrial contraction commences near the peak of the P-wave. In line with the pattern previously analyzed, the velocity following the peak of the P-wave displays an initial increase, followed by a decrease. Within the PQ interval, the process of the velocity change is characterized by a point of significant magnitude and a corresponding point of considerable minima. The point of great minima, typically located near the Q-wave, carries a negative value, denoted as *b*. In the modeling process, the position of the Q-wave is directly set to the location of the *c*-point. However, the position of the Q-wave is not fixed and depends on the lengths of the atrial systole (AS) phase $L_{asys}$, the QRS complex $L_{qrs}$, and the IVC phase $L_{ivc}$. The duration of the PQ interval, denoted as $L_{Pb}$, is calculated using the equation $L_{Pb}=L_{asys}-\left(L_{qrs}-L_{ivc}\right)$. A sinusoidal function is utilized to model the velocity change over this period, expressed as Equation (1).

(1) $$y_{Pb}\left[i\right]=\sin\left(2\pi f_{Pb}t\left[i\right]+\theta_{Pb}\right),$$

where the initial phase, $\theta_{Pb}$, is generated by a uniform distribution with a range of values in [π/12,π/4]. The frequency, $f_{Pb}$, is determined by Equation (2).

(2) $$f_{Pb}=\frac{1}{T}=\frac{1}{\alpha L_{Pb}},$$

where the coefficient, α, is generated by a uniform distribution with a range of values in [4/3,2]. The ventricular volume expands due to AS, with this expansion velocity being less than the maximum expansion velocity during the RPF, denoted as $v_{rpfp}$. The maximum velocity during the RPF period is generated by a uniform distribution within the range [0.02, 0.08]. Given $v_{rpfp}$, the peak atrial systole velocity $v_{afp}$ falls within the range $\left[0.2,0.5\right]\times v_{rpfp}$, with this ratio being randomly generated by a uniform distribution from [0.2, 0.5]. The velocity $v_{b}$ at the minima point Q is randomly generated by a uniform distribution, taking values in the range [−0.02,0.02]. Finally, the velocity of this phase is adjusted within the interval of the minimum value $v_{b}$ and the maximum value $v_{afp}$. During IVC, the ventricular volume is maintained constant, while the myocardium continues exhibiting motion. Based on actual radar measurements, the characteristics of myocardial motion are found to vary among individuals, but commonalities are observed. Typically, myocardial velocity is observed to gradually increase less than 0, reaching a local maximum point in the RS interval. This maximal point is conveniently denoted as *c*. Given that the location and size of the maximal point *c* are not fixed, *c* is randomly generated within the RS interval. The duration of the bc interval is denoted by $L_{bc}$. The maximal point has a velocity within the range of [−0.01,0.02], generated by a uniform distribution. To describe this process, a sigmoid function is employed, expressed as

(3) $$y_{bc}\left[i\right]=\frac{1}{1-e^{-k_{bc}t\left[i\right]}},$$

where $k_{bc}$ determines the rate of change. To ensure both the *b* and *c* points are within the saturation region of the sigmoid function, a condition is set such that $1/(1-\exp\left(-k_{bc}L_{bc}/2\right))$≥0.9, allowing for the calculation of the value of $k_{bc}$. The maximum velocity at point *c* is denoted by $v_{aop}$, taking values within the range of [0,0.02], randomly generated from a uniform distribution. The velocity is obtained by substituting the sampling time into Equation (3). Given the known minimum velocity $v_{b}$ and the maximum velocity $v_{aop}$, the values fit to the function are adjusted for magnitude. Upon the end of IVC, the ventricles are entered into a phase of rapid ejection initiated by the opening of the aortic and pulmonary valves. As the ventricular volume decreases rapidly, the rate of chest wall surface motion experiences a significant increase. This phenomenon is observable in the velocity of the CWM, as illustrated in Figure 3 and Figure 4. Examining the curve, it can be noticeable that the velocity decreases from point *c*, with a potential local minimum at point *J*. The duration of the cj interval, denoted as $L_{cj}$, can be calculated from the position of *c* and $L_{sj}$. Within the cj interval, an exponential decay function is employed to describe the process, expressed mathematically as:

(4) $$y_{cj}\left[i\right]=e^{-k_{cj}t\left[i\right]},$$

where $k_{cj}$ determines the decay rate. A condition is set such that the velocity within the cj interval decreases to 0.01, allowing for the determination of the value of $k_{cj}$. From the measured data, it is observed that the velocity at point *J* varies widely within the range of $\left[v_{rpe},0\right]$, where $v_{rpe}$ denotes the maximum motion velocity of the thoracic surface during the ejection period. From the analysis of the radar and camera data, this value was found to be within the range of 0.04 to 0.14. A random value is generated by uniformly distributing between 0.04 and 0.14 as $v_{rpe}$. The range of values for $v_{j}$ is set to be $\left[0.1,0.7\right]\times v_{rpe}$, and values within this range are randomly generated using a uniform distribution. The sampling time is determined based on the duration of this interval, and the velocity is obtained by substituting them into Equation (4). Finally, adjustments are made within this interval based on the maximum value $v_{aop}$ and the minimum value $v_{j}$.

Following point *J*, the velocity undergoes a rapid increase, followed by a deceleration, culminating in a localized peak at point *e*. Subsequently, the velocity decreases to a minimum value at $T_{start}$. After the peak at point *e*, the velocity experiences another rapid decline, featuring a minimum at $T_{start}$. The duration of the Je interval, which ranges between 0.02 s and 0.03 s, is randomly generated through a uniform distribution. To simulate the velocity change in the Je interval, the rising part of the Riley decay is employed, expressed as follows:

(5) $$y_{je}\left[i\right]=\frac{t_{je}\left[i\right]}{s_{e}^{2}}\cdot \exp\left(-\frac{t_{je}{\left[i\right]}^{2}}{2s_{e}^{2}}\right)$$

where the standard deviation $s_{e}$ equals $L_{je}$. The maximum velocity at *e* is denoted as $v_{e}$, with the requirement that $v_{e}\leq v_{j}$. Thus, the range of values for $v_{e}$ is set to $\left[v_{j},0.1\right]$, and $v_{e}$ is randomly generated from a uniform distribution. The sampling time is determined, and upon substituting it into Equation (5), the velocity within the interval is obtained. Finally, adjustments are made to the amplitude based on the minimum $v_{j}$ and maximum $v_{e}$ values.

In the segment from point *J* to $T_{end}$, two local minima are observed, occurring near the onset and the peak of the T-wave. For the ease of description, the first extreme point is labeled *f*, and the second extreme point is labeled *t*. Typically, the velocity near the initiation of the T-wave surpasses that at the peak of the T-wave. Given the duration of the ST segment, $L_{st}$, the SJ segment, $L_{sj}$, and the Je segment, $L_{je}$, the duration of the ef segment can be computed. An exponential decay model is employed to describe the ef segment, with the mathematical expression as Equation (5). The attenuation coefficient $k_{ef}$ is derived to ensure that the value at the end of the interval is below β, $e^{-k_{ef}L_{ef}}\leq \beta$. β is in the range of [0.001, 0.1]. From the measured data, it is evident that there is no consistent relationship between the velocity at *f* and the velocity at *J*. In some cases, the velocity at *J* exceeds that at *f*, and vice versa. To account for this variability, the range of values for the velocity at *f* is set to be $\left[0.5,1\right]\times v_{rpe}$. Utilizing a uniform distribution, a random velocity value within this range is generated, denoted as $v_{f}$. Upon confirming the sampling time, the velocity is determined by substituted it in Equation (5). Ultimately, the amplitude is adjusted based on the minimum $v_{f}$ and maximum $v_{e}$ values within the interval.

During the interval from the onset to the peak of the T-wave, the ejection velocity exhibits an increasing and then decreasing trend. Notably, there are an extreme value point *g* before the T-wave peak and an extreme value point *t* near the T-wave peak. To characterize this process, a sigmoid function is used for fitting. Initially, the location of the extreme point *g* needs to be determined. Since point *g* is in the first half of the T-wave and its position is not fixed, a uniform distribution in the interval $\left[0.2,0.3\right]\times L_{twave}$ is used to randomly generate the position of point *g*, where the length of the T-wave, $L_{twave}$, is calculated over $L_{sya}$, $L_{ivc}$, $L_{st}$. Next, the duration $L_{fg}$ of the interval from point *f* to point *g* is determined, which can be calculated from the position of point *g* and $L_{twave}$. According to the properties of the sigmoid function, if *f* and *g* are in the saturation region of the function, i.e., $1/1-e^{-k_{fg}t}\geq 0.99$, the steepness of the velocity $k_{fg}$ can be calculated. According to the results of the analysis of the measured data, the velocity $v_{g}$ at point *g* is expected to be greater than $v_{f}$. Therefore, the value of $v_{g}$ is randomly generated using a uniform distribution that takes values in the interval $\left[v_{f},0.01\right]$. Upon substituting the sampling time and $k_{fg}$ into Equation (3), the value of the velocity in the current interval is obtained. Finally, the amplitude is adjusted according to the minimum value $v_{f}$ and the maximum value $v_{g}$.

Near the peak of the T-wave, a minimum point *t* is observed. Its location is randomly determined by a uniform distribution with values in the interval $\left[0.4,0.6\right]\times L_{twave}$. The length from point *g* to point *t* is denoted as $L_{gt}$. Based on the analysis of the actual collected radar data, it was found that the velocity $v_{t}$ at point *t* is smaller than the velocity at point *f*. Therefore, the value of $v_{t}$ is generated from a uniform distribution with values in the range $\left[0.1,0.5\right]\times v_{f}$. Given the relatively slow rate of decay of the waveform, this process is modeled using a cosine signal, as in Equation (6). Here, the frequency $f_{gt}$ is determined by $L_{gt}$ and a factor $\alpha_{gt}$ in Equation (7). The range of values for $\alpha_{gt}$ is restricted to [1/6,3/5]. Finally, the value of $y_{gt}$ is adjusted by taking the last value of the fg segment as the maximum value and $v_{t}$ as the minimum value.

(6) $$y_{gt}=\cos\left(2\pi f_{gt}t\left[i\right]\right)$$

(7) $$f_{gt}=\frac{\alpha_{gt}}{L_{gt}}$$

During the final phase of systole, i.e., the interval from point *t* to $T_{end}$, the velocity is observed to gradually decrease to near zero. The end point is assumed to be located at *h*, considering that the location of *h* varies from person to person and is mainly located in the second half of the T-wave. The location of *h* is randomly generated using a uniform distribution taking values in the range $\left[0.75,1\right]\times L_{twave}$. At the same time, the velocity $v_{h}$ of *h* is randomly generated by a uniform distribution taking values in the range [−0.001,0.005]. Once the position of the point *t* with respect to *h* is determined, the duration $L_{th}$ can be calculated. To simulate the change in velocity over this interval, a sinusoidal signal is used. Let the period of the sinusoidal signal be $r_{th}L_{th}$, where the coefficients $r_{th}$ are randomly generated from a uniform distribution taking values in the range [3/2,2]. To ensure that the initial value of the sine is a minimum, the signal is phase-shifted by 3π/2. The mathematical expression is given in Equation (8). Finally, the value of $y_{th}$ is adjusted according to the interval of the maximum and minimum. This completes the modeling of the systolic phase of the cardiac cycle.

(8) $$y_{th}\left[i\right]=\sin\left(2\pi \frac{1}{r_{th}L_{th}}t\left[i\right]+\frac{3\pi }{2}\right)$$

Following the onset of ventricular diastole, the velocity during IVR exhibits a specific pattern of increasing and then decreasing, with distinct peaks and valleys. The magnitudes of these two values vary among individuals. Therefore, the peak velocity $v_{ivrp}$ is randomly generated from a uniform distribution taking values in the range [0.01,0.02], and the trough velocity $v_{ivrv}$ is randomly generated from a uniform distribution taking values in the range [−0.01,0] to produce the valley velocity value. Let the peak point be *i* and the trough point be *k*. Considering the skewed distribution property of the waveform in the hk region, a skewed distribution function is used to simulate the velocity change during this period. The mathematical expression is as follows:

(9) $$y_{hk}\left[i\right]=\frac{1}{\sqrt{2\pi \sigma^{2}}}e^{-\frac{{\left(t\left[i\right]-\mu \right)}^{2}}{2\sigma^{2}}}\left(1-\gamma {\left(\frac{t[i]-\mu }{\sigma }\right)}^{3}\right),$$

where μ is the peak position. It is within the IVR period, and its value is randomly generated from a uniform distribution with a range of $\left[1/3,1\right]\times L_{ivr}$. The standard deviation, σ, takes values in the range [0.01, 0.2] and is also generated from a uniform distribution. The parameter γ determines the direction of the skewness, less than 0 to the left and greater than 0 to the right. Considering that the measured data are mainly skewed to the left, its value is set in the range [−0.5,0.2], randomly generated by a uniform distribution. Due to the wide range of variation in the location of the valley point *k*, it may occur even in the filling zone. Therefore, the hk segment consists of three parts: the first part is from point *h* to $T_{end}$; the second part is $1/2\times L_{ivr}$; the third part is from the second half of the IVR period to half of the filling period. The position of *k* falls in the third part and is randomly generated by a uniform distribution. Finally, the sampling time, mean μ, standard deviation σ, and γ are substituted into Equation (9) for the calculation. Adjustments are made for the minimum value $v_{ivrv}$ and the maximum value $v_{ivrp}$ in the interval. This completes the modeling of the IVC phase of the cardiac cycle.

During the filling phase of the heart, the ventricles gradually increase in volume as they fill with blood. This results in a tendency for the velocity at the chest wall surface to initially increase and, subsequently, decrease. To model this process, the Rayleigh distribution (RD) is used to describe the change in velocity at the chest wall surface, as shown in Equation (5). The variance, denoted as $\sigma^{2}$, determines the shape of the distribution and the location of the peak. It takes values in the range $\left[0.3,0.7\right]\times L_{rpf}$ and is generated by a uniform distribution. However, it is also constrained to lie between [0.03, 0.06]. The maximum chest wall surface velocity during the RPF phase, denoted as $v_{rpf}$, is smaller than the maximum chest wall surface velocity during the fast ejection phase. The peak velocity $v_{rpf}$ is set to take a value in the range [0.02, 0.08] and is randomly generated from this range using a uniform distribution. The sampling time is determined based on the position of point *k*, $L_{rdf}$, and $L_{rpf}$, and the velocity is obtained by substituting these into Equation (5). Finally, the values are adjusted according to the maximum value $v_{rpf}$ and the minimum value of 0 within the interval. The chest wall motion (CWM) velocity for the entire cardiac cycle is simulated by the above steps, allowing us to obtain the CWM velocity curve for one cardiac cycle. The CWM parameters associated with the model are listed in Table 2. This completes the modeling of the diastolic phase of the cardiac cycle.

#### 2.2.3. Integrating the Velocity Profile Yields a Displacement Profile

**Figure 5:**
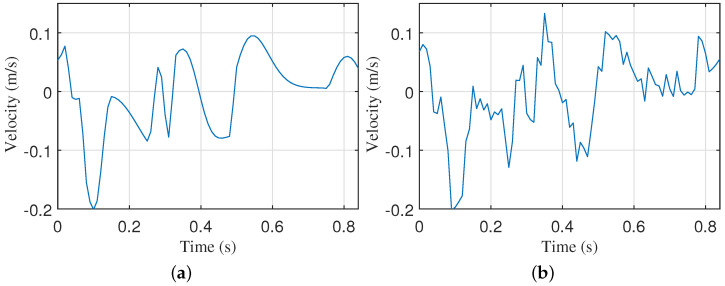
Velocity of CWM generated by proposed model. (**a**) Velocity without noise. (**b**) Velocity with 10 dB Gaussian noise.

In practice, the CWM exhibits significant complexity and varies among individuals. To bring the simulation results closer to real-world scenarios, Gaussian white noise is introduced to the simulated signal. The simulated velocity of the CWM with added noise is shown in Figure 5. The velocity profile of the CWM effectively encompasses the cardiac cycle, exhibiting a shift from approximately 0 during IVC to near 0 at the conclusion of atrial systole. By performing the integration of the velocity profile, the displacement profile is derived. This approach allows for a more realistic simulation of the cardiac cycle, accounting for the inherent variability and complexity of the CWM.

#### 2.2.4. Long Sequence Generation

In practical scenarios, data collection typically spans a period of time. As a result, data of an arbitrary duration can be generated by concatenating the displacement curves from multiple cardiac cycles. However, even during periods of rest, the cardiac cycle exhibits slight variations between each cycle. These variations are characterized by HRV, with the normal range for an individual being [−0.1, 0.1]. To model this phenomenon, the mean of the variability is set to 0 and a uniform distribution is utilized to randomly generate standard deviations within the range of values [−0.015, 0.015]. Heart rate variability for each cardiac cycle is generated by constructing a Gaussian distribution. The beat–beat interval (BBI) is finally obtained by HRV. The length of the cardiac cycle is varied by means of random deletion or interpolation so that the cycle_len of each cardiac cycle is the same as the BBI after the introduced HRV. The CWM displacement is then obtained by integrating the whole long sequence. It is worth noting that The velocities during each period are also unequal due to the unequal duration of systole and diastole. This leads to the displacement curves obtained by integration not being equal at the beginning and the end. That is, the amplitude of diastolic motion is not equal to the amplitude of systolic motion. This results in a tendency for the displacements obtained by integration to be skewed upwards or downwards. A polynomial regression [30] is used to correct for this tendency. Suppose the signal without tendency is x(t), the approximate function of the tendency is p(t), and the observed signal is y(t). p(t) can be expressed as Equation (10).

(10) $$p\left(t\right)=a_{0}+a_{1}t+a_{2}t^{2}\cdots +a_{k}t^{n}=\sum_{i=0}^{n}a_{n}t^{n},$$

where $t_{i}$ denotes time and $y(t_{i})$ is magnitude. The number of points is *M*; *i* is in the range of 1≤i≤M. Ignoring the noise, let the original signal be y(t)=x(t)+p(t). As long as the errors of $y(t_{i})$ and $p(t_{i})$ are minimized, $p(t_{i})$ is close to the true trend. Using the MSE as the evaluation criterion:

(11) $$S\left(a_{0},a_{1},...,a_{n}\right)=\frac{1}{M}\sum_{i=1}^{M}{\left[p\left(t_{i}\right)-y\left(t_{i}\right)\right]}^{2}$$

Find the first-order derivative of $a_{i}$ in Equation (11), and set the derivative equal to 0. The coefficients of each coefficient $a_{i}$ in p(t) can be determined. It was experimentally verified that it is more appropriate to use 3rd-order polynomial regression for trajectory fitting, while ensuring the fitting approximation and computational effort.

## 3. Experimental Results and Discussion

### 3.1. Simulated Heartbeat Waveform Analysis

#### 3.1.1. Waveforms at Different Parameters

**Figure 6:**
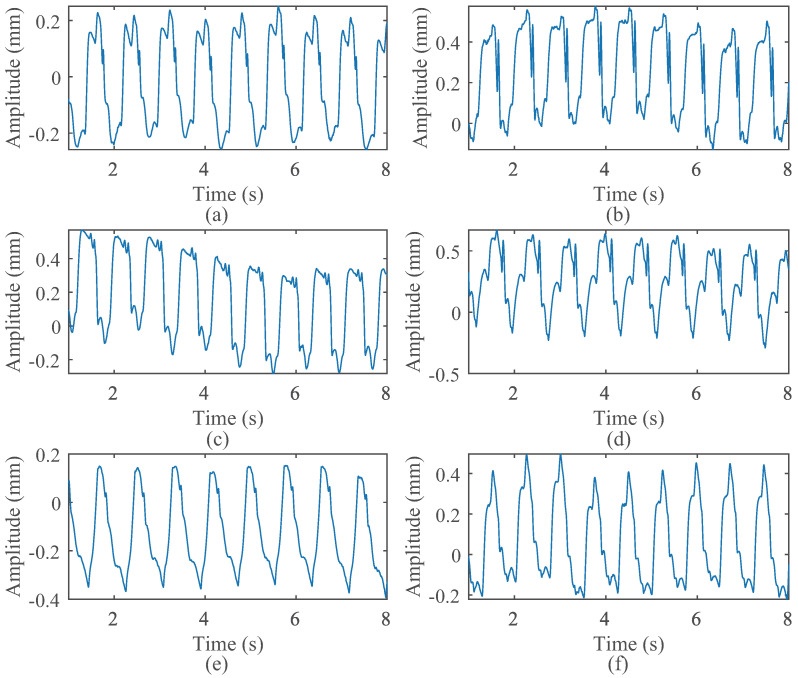
CWM displacement. (**a**–**f**) are waveform generated by proposed model under different parameters.

Table 2 comprises parameters that are autonomously generated for each trial in accordance with the specified distribution. This process, in turn, yields a chest wall velocity profile for a single cardiac cycle. By integrating the velocity with noise, the displacement is derived. Displacement curves from each cardiac cycle are concatenated, taking into account the effect of heart rate variability, thereby resulting in a lengthy sequence. Figure 6 depicts 8 s simulated CWM waveforms, each with unique parameter settings. Despite the differences among these waveforms, certain similarities are discernible. The primary reason for these similarities lies in the process of segmented modeling, wherein the mathematical function utilized for each segment remains consistent. When the values of the randomly generated parameters are similar within the same segmentation, the resulting model exhibits approximation. Even with the inclusion of relatively large white noise, the overall trend retains its similarity. Moreover, the variability among the waveforms obtained with different parameters is highly noticeable. It is apparent that, in addition to the fundamental frequency, higher-order harmonics are also present, with their intensity decreasing as the order increases.

#### 3.1.2. Spectrum Analysis

**Figure 7:**
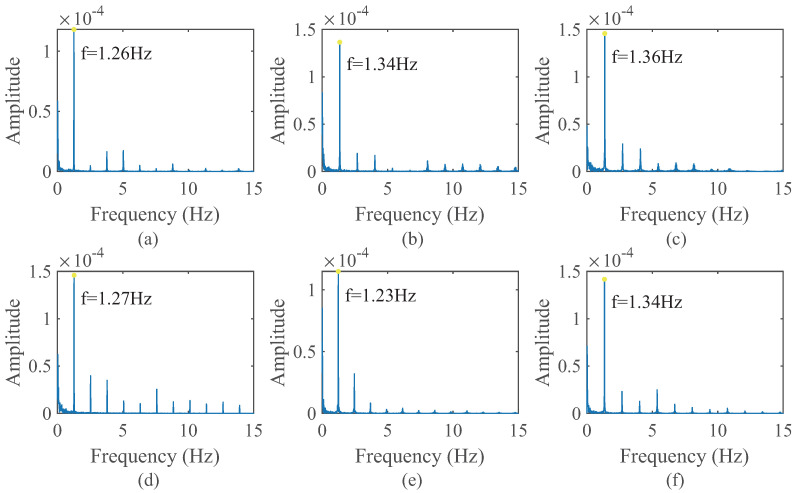
Amplitude Spectrum of simulated signal. (**a**–**f**) are the spectrum of signal in Figure 6.

In the process of extracting vital signs from radar signals, the subtle waveform of the heartbeat is frequently obscured by the respiratory waveform. Once the heartbeat waveform is obtained through filtering, spectral analysis is typically employed to determine the heartbeat frequency. Consequently, it is imperative to validate the spectral characteristics of the simulated heartbeat waveform. Spectral analysis was conducted on the data generated in the preceding subsection. Specifically, a Hamming window was first applied to the simulated heartbeat curve, followed by performing a Fast Fourier Transform (FFT). The resulting spectrogram is depicted in Figure 7. It is evident that, in addition to the fundamental frequency, higher-order harmonics are also present, with their intensity diminishing as the order increases.

### 3.2. Comparisons with Recorded Radar Signals and VICON Signals

#### 3.2.1. Qualitative Inorganic Analysis Based on Waveform

The proposed model is an empirical model based on real signals. To enable the model to generate more diverse data, we introduced many random variables into the model design. By inputting the characteristic parameters of the real signal, the model should be able to generate data waveforms that approximate the real data. Therefore, we compared the generated data waveforms with the real data waveforms given different parameters (extracted from the real signal).

**Figure 8:**
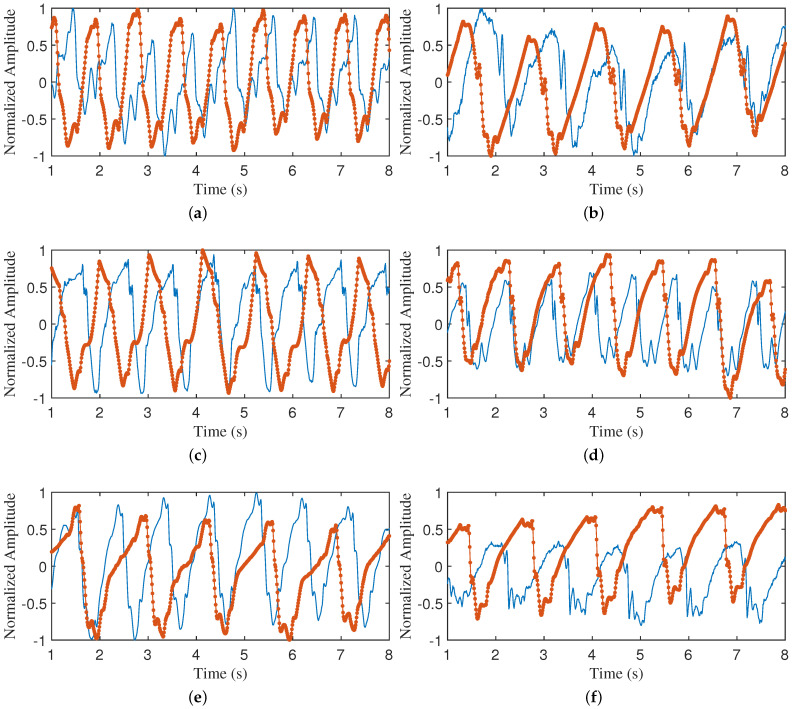
Comparison of simulated waveform and radar measured CWM waveform. (**a**–**f**) are simulated waveform generated by given specific parameters and radar-measured CWM waveform, respectively. 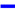, radar measured CWM waveform; 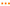, simulated waveform.

**Figure 9:**
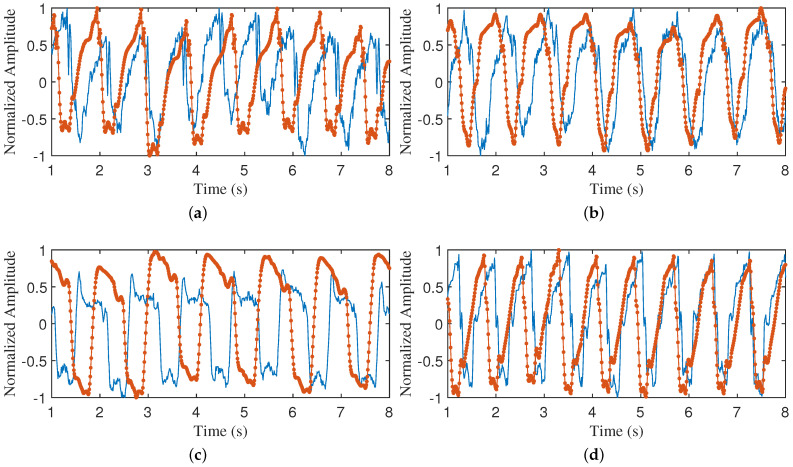
Comparison of simulated waveform and VICON-measured CWM waveform. (**a**–**d**) are simulated waveform generated by given specific parameters and VICON-measured CWM waveform, respectively. 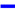, VICON CWM waveform; 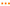, simulated waveform.

The data were selected from a dataset collected from real scenarios, with an 8 s segment of data intercepted for each individual. For each data segment, the cardiac cycle, systole, diastole, and BBI were calculated. Upon entering these parameters into the model, a simulated chest wall displacement profile was obtained. To visually compare the differences between the simulated and real signals, these waveforms were directly compared and qualitatively analyzed. As can be observed from Figure 8 and Figure 9, the proposed model is capable of simulating the real CWM curve to a high degree. It is noteworthy that the proposed model can fit the real CWM curves on a case-by-case basis, despite the differences in the real CWM curves of different individuals. A quantitative analysis will be conducted subsequently.

#### 3.2.2. Comparison of Spectrum between Simulated Signal and Real Signal

**Figure 10:**
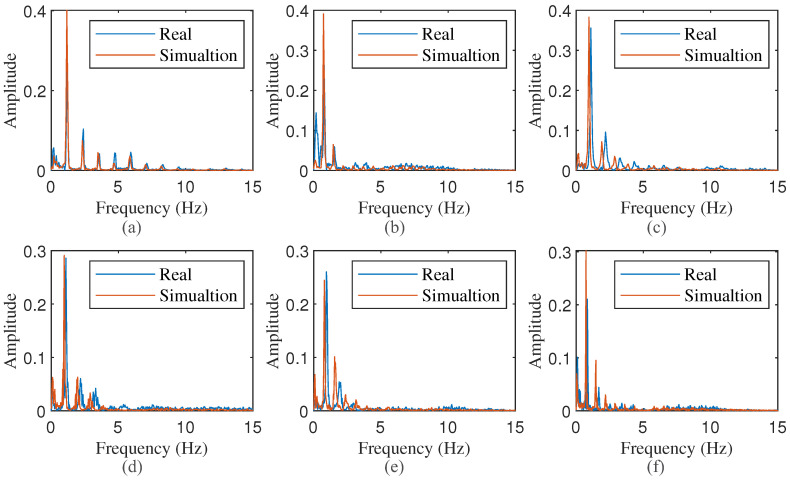
Spectrum comparison of simulated waveform generated by given specific parameters and radar-measured CWM waveform. (**a**–**f**) are the spectrum of signal in Figure 8 respectively. 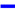 spectrum of radar-measured CWM waveform; 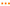 spectrum of simulated waveform.

**Figure 11:**
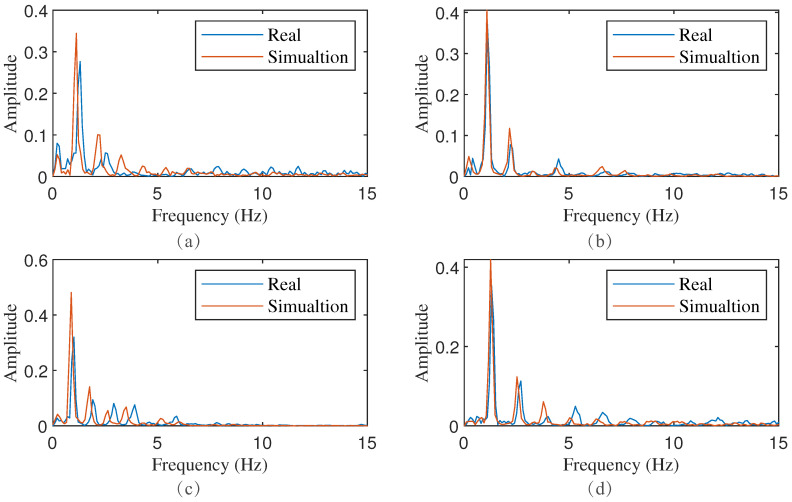
Spectrum comparison of simulated waveform generated by given specific parameters and VICON-measured CWM waveform. (**a**–**d**) are the spectrum of signal in Figure 9 respectively. 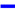, spectrum of VICON CWM waveform; 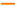, spectrum of simulated waveform.

In the preceding subsection, waveforms, which were generated under specific parameters, were analyzed for their qualitative similarity to the actual CWM as measured by radar and the VICON system. This subsection further compares the magnitude spectrum of these waveforms to assess their similarity in the frequency domain. The calculation of the magnitude spectrum adheres to the previously outlined method. Figure 10 and Figure 11 display the magnitude spectrum of the corresponding waveforms depicted in Figure 8 and Figure 9, respectively. The comparison results revealed that the simulated signal spectrum, generated by our proposed model, bears a high degree of similarity to both the CWM spectrum obtained from radar and the VICON system. Notably, the maximum frequency components and their corresponding harmonics overlap almost exactly. These findings suggest that, by incorporating the features extracted from the actual observed CWM as parameters into our proposed model, not only can simulated waveforms with high similarity be obtained in the time domain, but highly similar results can also be realized in the frequency domain.

#### 3.2.3. Quantitative Analysis Based on DTW and RMSE

The DTW algorithm is widely used in many fields and is recognized as an excellent similarity measure. DTW measures the similarity of two different sequences, especially when the sequence lengths do not coincide. The algorithm first computes a matrix of Euclidean distances between points in the two sequences, and then, the shortest path from the upper-left corner of the matrix to the lower-right corner that minimizes the sum of the elements on the path is found. The distance of this minimum sum is the DTW distance between the two sequences. In general, a smaller DTW distance indicates a higher similarity between the two sequences. The formula for the DTW algorithm is given in Equation (12):

(12) $$D\left(i,j\right)=d\left(A_{i},B_{j}\right)+\min\left(D\left(i-1,j-1\right),D\left(i-1,j\right),D\left(i,j-1\right)\right),$$

where D(i,j) denotes the DTW distance from the first element of *A* to the *i*th element and from the first element of *B* to the *j*th element. $d(A_{i},B_{j})$ denotes the distance between $A_{i}$ and $B_{j}$, which is generally the Euclidean distance. min(D(i−1,j−1),D(i−1,j),D(i,j−1)) denotes the cumulative distance of the optimal path from the upper left corner to the position (i,j). When the sequence lengths are the same, the RMSE can also be used to evaluate the similarity of two signals. The RMSE measures the average degree of deviation between the predicted value and the true value. The formula for the RMSE is given in Equation (13):

(13) $$RMSE=\sqrt{\frac{1}{n}\sum_{i=1}^{n}{\left(s_{i}-x_{i}\right)}^{2}},$$

where *n* is the number of data, $s_{i}$ is the value of the CWM displacement captured by the radar during breath hold, and $x_{i}$ is the value of the simulated CWM displacement. The RMSE is always non-negative, and a value of 0 indicates that the predicted value is exactly the same as the true value. In general, a smaller RMSE indicates the superior performance of the prediction model.

The simulated waveform and the actual measurement waveform differ in terms of start time and length. Therefore, the initial 5 s signal segment is extracted from the simulated waveform. This segment is then used to calculate the distance using DTW and the RMSE with an equal-length segment of the real waveform. A sliding window with a step size of two and a total length of 5 s is used to analyze the intercepted real signal segment. The DTW distance and RMSE are calculated between the simulated signal and multiple intercepted real signal segments. The smallest DTW distance and RMSE values are selected as a measure of the similarity between the current simulated signal and the real signal. The similarity of the CWM waveforms generated based on the parameters and the real CWM signals is listed in Table 3.

### 3.3. Comparison with Other Methods

Singh et al. described the chest wall motion induced by a heartbeat directly as the rhythmic behavior of the sinusoatrial (SA) node [23]. A relaxation oscillation system is used to express their model in Equation (14). This model is also named as nonlinear van der pol model.

(14) $$\frac{d^{2}x}{dt^{2}}-\alpha \left(1-x^{2}\right)\frac{dx}{dt}+\omega^{2}x=0$$

The differential equation is resolved with an initial value of [0,1] to obtain the result. The evident pulsation of human heartbeat motion on the chest wall is contrasted with a mere sinusoidal pattern, as presented in [31,32]. A Gaussian pulse-train modeling of the heartbeat was proposed by Nosrati et al. [25]. The model is given by Equation (15).

(15) $$x\left(t\right)=\sum_{n}ae^{-\frac{{\left(t-Tn\right)}^{2}}{2c^{2}}},\;T_{n}=T_{1},T_{2},T_{3},\ldots ,$$

where *a* is the normalization factor, *c* is the pulse width, and $T_{n}$ is the time interval between two consecutive pulses *n* and n−1. This model, like our proposed model, allows the customization of $T_{n}$, which determines the cardiac cycle duration. A chest wall displacement profile for the model is obtained with a=0.2, c=0.1, and $T_{n}$ generated by a Gaussian distribution.

Nosrati et al. [24] also proposed to use an improved Gaussian pulse model to simulate the chest wall acceleration acquired by the accelerator and, then, a quadratic integration of the simulated acceleration to obtain the displacement. The expression can be found in Equations (16) and (17):

(16) $$x\left(t\right)=g\left(t\right)\cdot e^{-\frac{{\left(t-b\right)}^{2}}{c}}$$

(17) g(t)=η·cos(ωt+γ·sin(Ωt)),

where *t* is the number of sampling points and *b* and *c* are the constants. The peak position depends on the conditioning parameter, ω and Ω. The peak amplitude depends on η and γ. The parameters are listed in Table 4. An acceleration profile, fit to the cardiac cycle, is obtained. Following a quadratic integration of the acceleration curve, the chest wall displacement for one cycle is derived. A Gaussian distribution is used to generate the cycle lengths for which the HRV is not zero.

**Figure 12:**
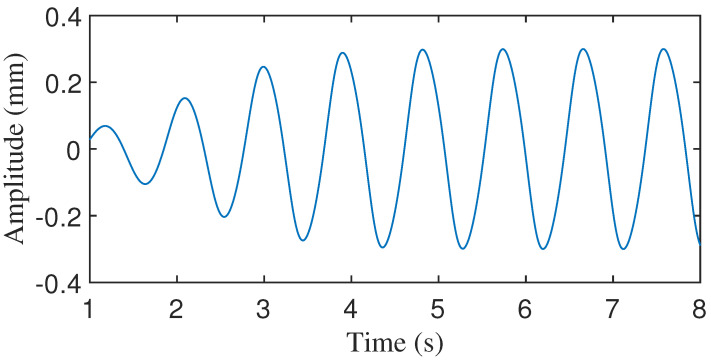
CWM generated by van der Pol model.

**Figure 13:**
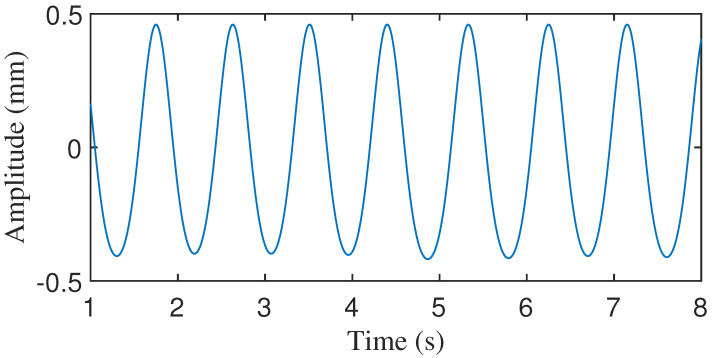
CWM generated by Gaussian pulse-train model.

**Figure 14:**
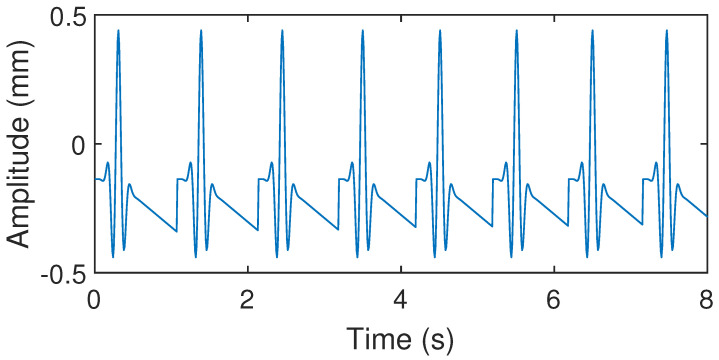
CWM generated by improved Gaussian pulse-train model.

The mean cardiac cycle and BBI were extracted from the radar and VICON system acquisition data in the previous subsection. These parameters were applied to the three models described above. The CWM waveforms generated by these models are shown in Figure 12, Figure 13 and Figure 14. These waveforms differ significantly from the CWM waveforms generated by our proposed model (Figure 6) and the actual CWM waveforms (Figure 8 and Figure 9). The relaxation oscillation model produces a sinusoidal signal, while the Gaussian pulse-train model generates a series of wide Gaussian pulses. The waveform produced by the improved Gaussian pulse model resembles an acceleration rather than a chest wall displacement.

The parameters extracted from the data collected from multiple human bodies in the previous subsection were applied to each model to simulate the generation of CWM data. The same method as in the previous subsection was used to calculate the similarity between the simulation-generated CWM waveforms and the real CWM waveforms detected by the radar and VICON system. Since many parameters in this model are randomly generated, the waveforms produced by these model are different, even though the features extracted from the real data are the same for each input.

**Figure 15:**
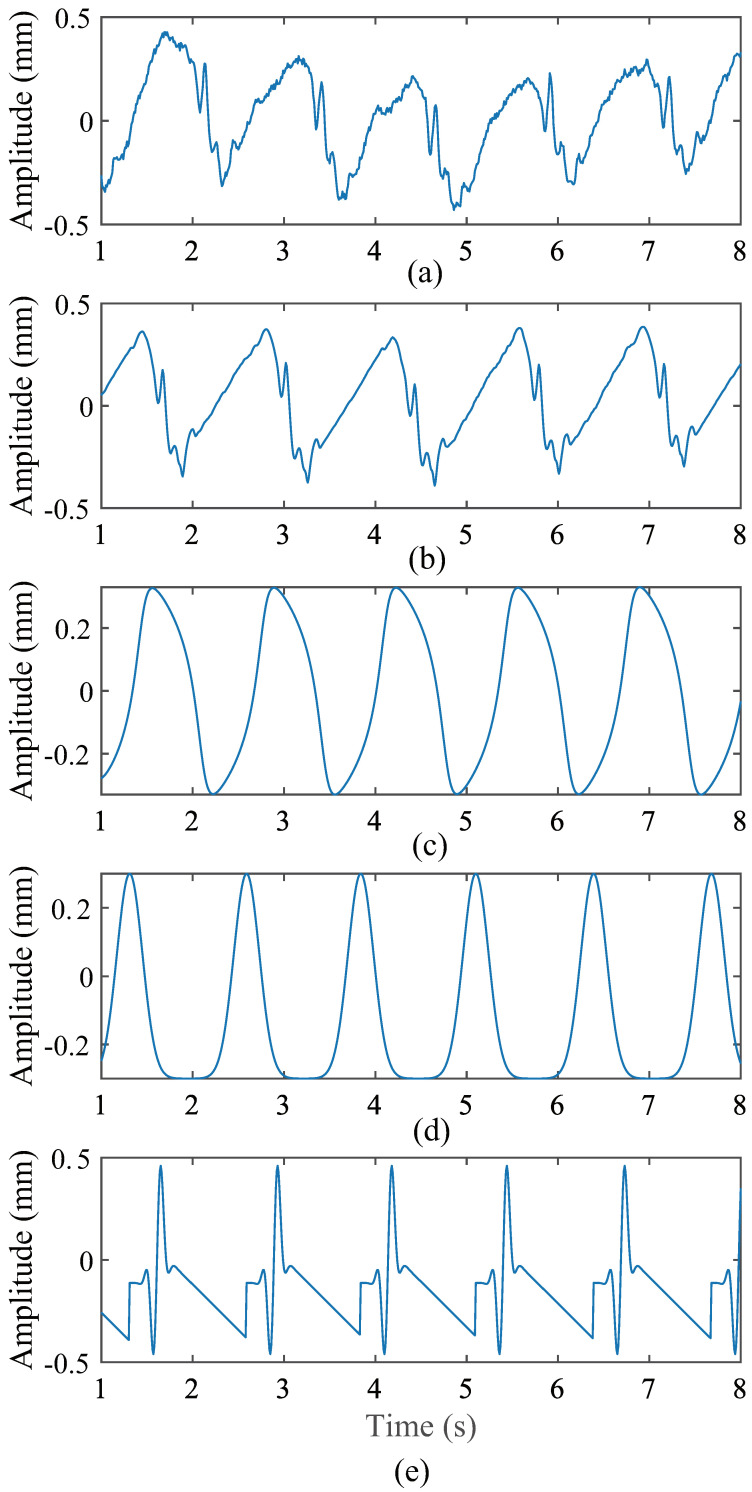
Comparison of CWM waveforms of subject 5 and waveforms of each simulation. (**a**) CWM detected by radar. (**b**) The simulated waveform produced by the proposed model. (**c**) The simulated waveform produced by the model proposed in [23]. (**d**) The simulated waveform produced by the model proposed in [24]. (**e**) The simulated waveform produced by the model proposed in [25].

**Figure 16:**
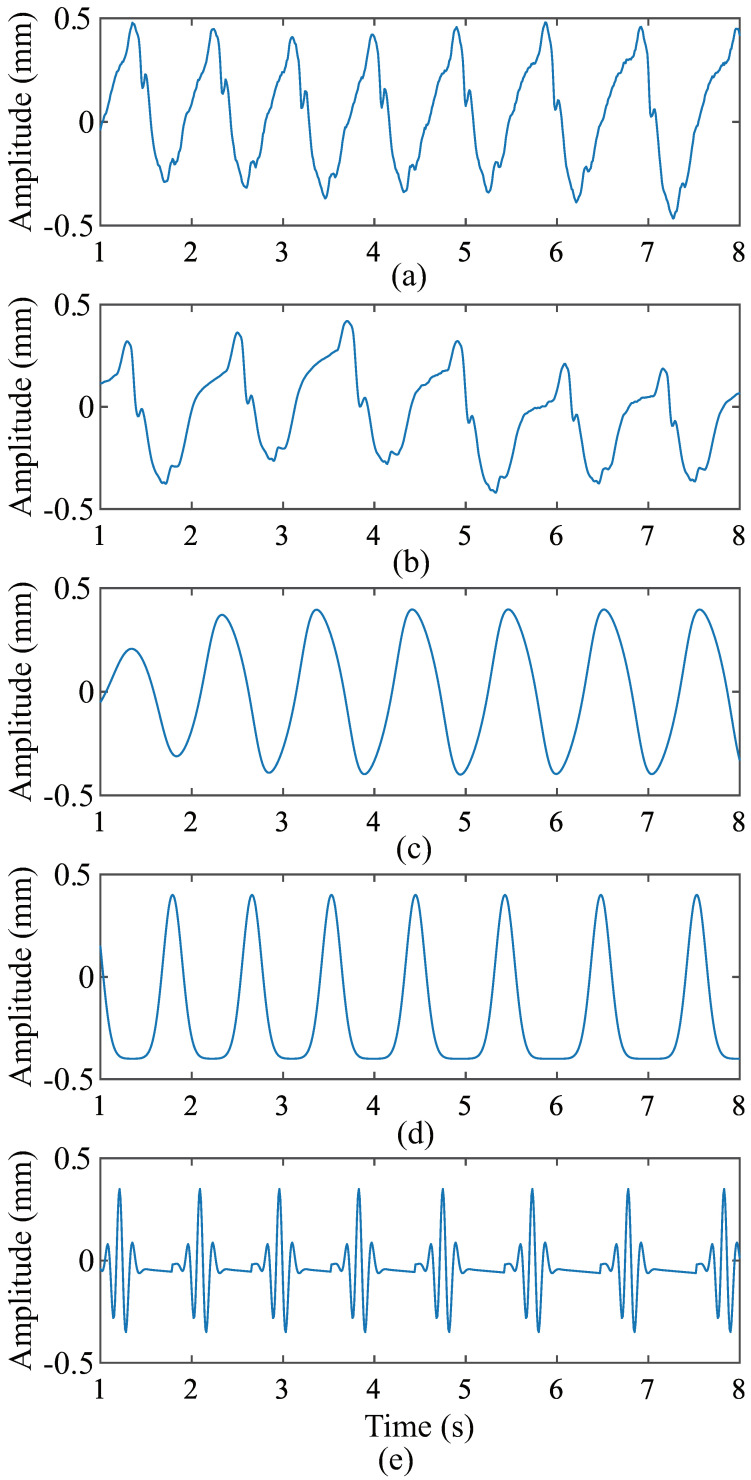
Comparison of CWM waveforms of subject 12 and waveforms of each simulation. (**a**) CWM detected by radar. (**b**) The simulated waveform produced by the proposed model. (**c**) The simulated waveform produced by the model proposed in [23]. (**d**) The simulated waveform by the model proposed in [24]. (**e**) The simulated waveform produced by the model proposed in [25].

To minimize the error, five waveforms were produced and the distances calculated, and the average distance was taken. Table 5, Table 6, Table 7 and Table 8 list the results of our proposed method and other related methods, respectively. The data in these two tables show that, in most cases, the distance value between our proposed method and the actual CWM waveforms detected by the radar is smaller, indicating a higher degree of similarity. For both DTW distance and RMSE, the simulation data generated by our model based on the data collected by the VICON system have the highest similarity to the actual CWM waveforms detected by the VICON system. However, there exist some individual cases where our model did not perform well in the distances computed by DTW and the RMSE, such as subject 5 in Table 5 and subject 12 in Table 7. The actual CWM waveforms of individuals 5 and 12 were compared with the CWM waveforms generated by our proposed model and the other three models, as shown in Figure 15 and Figure 16, for further analysis of these cases. In Figure 15b, the diastolic velocity of the proposed model is less than the actual velocity during diastole, resulting in large differences in the waveform. In addition to this, the details of the waveform of our simulated data are closer to the real signal. The waveform with the smallest distance calculated by DTW from the real signal loses almost all details. Additionally, DTW is more sensitive to noise and outliers, which is worth noting. A CWM with many details that may be mistaken as noise when calculating DTW was generated by our proposed model, potentially introducing errors. Despite not producing the smallest distances when using DTW, the waveform shape of our model is the closest to the real CWM. In Figure 16, the RMSE of the waveforms in Figure 16b and Figure 16a is larger than the RMSE of the waveforms in Figure 16a,d. However, the waveform in Figure 16b more closely resembles a heartbeat signal, while the waveform in Figure 16d is merely a regular burst. The reason for the large RMSE is probably that the BBI in Figure 16b is more distorted than the BBI in Figure 16d. Overall, the signals generated by our proposed model are more similar to the real signals, both in terms of the distance calculated by DTW and the distance calculated by the RMSE. Our proposed model outperforms than other methods.

## 4. Discussion

### 4.1. Applicability of the Proposed Model

Given that the ages of the participants in the two datasets are 25.36±2.93[26] and 30.9±9.9 [3], these datasets do not include data from children and elderly individuals. We extracted features from these data and then built a model. The proposed model, limited by the age distribution of the participants, may not accurately simulate chest wall motion in children and elderly individuals. In addition, the model relies on data from healthy individuals. The model is primarily applicable to healthy individuals or those with relatively intact circulation. The data generated by the model may not correlate well with individuals at risk for cardiac arrest or with very weak breathing and heartbeat. Further optimization of the model is required. The model’s applicability to different populations will improve as more data are collected from children, elderly individuals, and those with cardiovascular disease.

### 4.2. Limitation of the Proposed Model

The proposed piecewise CWM model segmentally simulates heartbeat-induced chest wall motion. Although the model introduces a multitude of random parameters to simulate a wide range of data, it has limitations in accurately simulating the actual CWM. This is due to the differences in the signals of each segment and the significant variances between different individuals. Additionally, the model, which is constructed based on the summarizing features extracted from the data, still exhibits differences in details after the introduction of noise and random variables, even though the generated waveforms maintain a certain degree of similarity. Deep learning algorithms [33,34] are widely applied in areas such as time series generation, prediction, and performance prediction [35]. However, the question of how to train models to produce the desired CWM signals with limited data remains an issue that warrants in-depth exploration.

### 4.3. Potential to Detect Cardiovascular Diseases

In patients with cardiovascular disease, chest wall displacement induced by heartbeats can be distinguished. Given that the currently proposed model is primarily constructed based on data from healthy individuals, it may hold potential for detecting cardiovascular disease by comparing the differences in chest wall motion between healthy individuals and patients with cardiovascular disease. However, to validate this potential, more data from patients with cardiovascular disease need to be collected and analyzed. Furthermore, more in-depth research needs to be conducted in close collaboration with experts in the cardiovascular field.

## 5. Conclusions

This study proposes a piecewise CWM model for the field of radar-based noncontact vital sign detection. The model is based on the measured features of the VICON IR camera and radar data and focuses on heartbeat-induced CWM, excluding respiratory interference. The results demonstrate that simulated CWM data from multiple individuals can be generated, effectively addressing the issue of limited radar datasets in the context of deep learning methods. With the simulated data, various deep learning methods can be employed to tackle problems such as estimating heart rate under significant respiratory disturbances and distinguishing between respiratory and heartbeat signals. Additionally, refined CWM models exhibit potential for the detection of cardiovascular diseases.

## Figures and Tables

**Table 1:** Information of all the subjects.

Dataset	ID	Age	Sex	Height (cm)	Weight (kg)	BMI
VICON data	1	24	M	173	62	20.7
	2	26	M	168	63	22.3
	3	22	M	168	61	21.6
	4	28	M	175	70	22.9
24 GHz CW data	1	28	F	178	59	18.6
	2	27	F	173	93	31.1
	3	49	F	172	63	21.3
	4	40	M	184	74	21.9
	5	24	M	186	78	22.5
	6	24	M	193	86	23.1
	7	35	F	153	44	18.8
	8	49	F	167	85	30.5
	9	23	F	172	68	23.0
	10	27	M	186	69	19.9
	11	26	M	183	82	24.5
	12	29	M	190	94	26.0
	13	25	M	186	82	23.7
Mean		29.8	-	176.9	72.5	23.1
STD		8.2	-	9.9	13.0	3.4

M: male, F: female, BMI: body mass index (kg/m^2^), STD: standard deviation.

**Table 2:** Parameters in the proposed heart model.

Parameters	Symbol	Value	Ways to Generate Values
Cardiac cycle	cycle_len	[0.58,1.07] ms	Gaussian distribution,
			μ = 0.82, σ = 0.08
Systolic/diastolic ratio	*r*	[0.7,0.9]	Uniform distribution
Duration of systole	$L_{sys}$	[0.247,0.53] ms	Calculated by cycle_len and *r*
Duration of diastole	$L_{dia}$	[0.3,0.624] ms	Calculated by cycle_len and *r*
Duration of QRS complex	$L_{qrs}$	$\left[1.8,2.5\right]\times L_{ivc}$, <120 ms	Uniform distribution
Duration of IVC	$L_{ivc}$	$\left[0.9,0.13\right]\times L_{sys}$ ms	Uniform distribution
Duration of SJ	$L_{sj}$	[0.01,0.03] ms	Uniform distribution
Duration of ST segment	$L_{st}$	[0.07,0.09] ms	Uniform distribution
Duration of IVR	$L_{ivr}$	$\left[0.9,0.13\right]\times L_{dia}$ ms	Uniform distribution
Duration of atrial systole	$L_{asys}$	$\left[0.16,0.22\right]\times L_{dia}$ ms	Uniform distribution
Maximum velocity of CWM during RPF	$v_{rpfp}$	[0.02,0.08] m/s	Uniform distribution
Maximum velocity of CWM during AS	$v_{afp}$	$\left[0.2,0.5\right]\times v_{rpfp}$ m/s	Uniform distribution
Minimum velocity of CWM during AS	$v_{b}$	[−0.02,0.02] m/s	Uniform distribution
Maximum velocity of CWM during IVC	$v_{aop}$	[−0.01,0.02] m/s	Uniform distribution
Maximum velocity of CWM during RPE	$v_{rpe}$	[−0.04,−0.14] m/s	Uniform distribution
The velocity at point *J*	$v_{j}$	$\left[0.1,0.7\right]\times v_{rpe}$ m/s	Uniform distribution
Duration of the Je interval	Je	[0.02,0.03] ms	Uniform distribution
The velocity at point *e*	$v_{e}$	$\left[v_{j},0.1\right]$ m/s	Uniform distribution
The velocity at point *f*	$v_{f}$	$\left[0.5,1\right]\times v_{rpe}$ m/s	Uniform distribution
The velocity at point *g*	$v_{g}$	$\left[v_{f},0.01\right]$ m/s	Uniform distribution
The velocity at point *t*	$v_{t}$	$\left[0.1,0.5\right]\times v_{f}$ m/s	Uniform distribution
The velocity at point *h*	$v_{h}$	[−0.001,0.005] m/s	Uniform distribution
Maximum velocity of CWM during IVR	$v_{ivrp}$	[0.01,0.02] m/s	Uniform distribution
Minimum velocity of CWM during IVR	$v_{ivrv}$	[−0.01,0] m/s	Uniform distribution
HRV	-	[−0.1,0.1]	Gaussian distribution,
			μ = 0, σ = 0.015
Amplitude	*a*	[0.5,1.1] mm	Uniform distribution

**Table 3:** Distance between the data generated by proposed model and the real data using DTW and RMSE.

Dataset	ID	DTW	RMSE
VICON data	1	53.80	0.51
	2	29.77	0.31
	3	38.54	0.45
	4	32.55	0.31
24 GHz CW data	1	34.31	0.35
	2	66.70	0.47
	3	55.58	0.75
	4	37.60	0.31
	5	55.32	0.62
	6	32.99	0.29
	7	21.31	0.27
	8	42.85	0.39
	9	28.34	0.46
	10	39.31	0.45
	11	38.03	0.62
	12	41.69	0.58
	13	28.73	0.23

**Table 4:** Parameters for improved Gaussian pulse-train model.

Symbol	Parameters	Value
*t*	Sample sequence	0:t:cycle_len
*b*	Constant	50
*c*	Constant	[0.01, 0.02]
ω	Conditioning parameter	0.2
γ	Conditioning parameter	0.1
Ω	Conditioning parameter	0.3
η	Conditioning parameter	0.5

**Table 5:** Distance between the data generated by each model and the real data using DTW.

Subject	Ref. [23]	Ref. [24]	Ref. [25]	Our Work
1	65.13	97.66	75.29	**34.31**
2	74.32	95.68	71.78	**66.70**
3	62.89	93.68	82.3	**55.58**
4	53.29	85.89	72.59	**37.60**
5	74.81	44.82	82.74	55.32
6	68.39	68.44	65.29	**32.99**
7	71.19	52.18	76.51	**21.31**
8	72.37	142.43	76.61	**42.85**
9	78.16	99.23	73.92	**28.34**
10	53.97	61.42	80.42	**39.31**
11	58	44.88	72.34	**38.03**
12	48.07	63.98	85.69	**41.69**
13	97.91	102.86	66.16	**28.73**

**Table 6:** Distance between the data generated by each model and the VICON data using DTW.

Subject	Ref. [23]	Ref. [24]	Ref. [25]	Our Work
1	88.80	78.55	79.15	**53.80**
2	79.31	50.84	90.34	**29.77**
3	55.35	90.18	83.50	**38.54**
4	79.10	70.17	84.26	**32.55**

**Table 7:** Distance between the data generated by each model and the real data using the RMSE.

Subject	Ref. [23]	Ref. [24]	Ref. [25]	Our Work
1	0.49	0.69	0.54	**0.35**
2	0.65	0.71	0.47	**0.47**
3	0.65	0.76	0.68	0.75
4	0.66	0.79	0.53	**0.31**
5	0.49	0.5	0.64	0.62
6	0.56	0.58	0.53	**0.29**
7	0.51	0.62	0.52	**0.27**
8	0.66	0.79	0.57	**0.39**
9	0.52	0.99	0.68	**0.46**
10	0.6	0.6	0.51	**0.45**
11	0.59	0.68	0.62	0.62
12	0.58	0.65	0.56	0.58
13	0.68	0.57	0.4	**0.23**

**Table 8:** Distance between the data generated by each model and the VICON data using DTW.

Subject	Ref. [23]	Ref. [24]	Ref. [25]	Our Work
1	0.49	0.59	0.56	0.51
2	0.49	0.42	0.64	**0.30**
3	0.64	0.67	0.54	**0.45**
4	0.57	0.56	0.63	**0.31**

## Data Availability

The data presented in this study are available on request from the corresponding author.

## References

[1] Droitcour A.D., Boric-Lubecke O., Lubecke V.M., Lin J., Kovacs G.T. (2004). Range correlation and I/Q performance benefits in single-chip silicon Doppler radars for noncontact cardiopulmonary monitoring. IEEE Trans. Microw. Theory Tech..

[2] Kim J.Y., Park J.H., Jang S.Y., Yang J.R. (2019). Peak Detection Algorithm for Vital Sign Detection Using Doppler Radar Sensors. Sensors.

[3] Schellenberger S., Shi K., Steigleder T., Malessa A., Michler F., Hameyer L., Neumann N., Lurz F., Weigel R., Ostgathe C. (2020). A dataset of clinically recorded radar vital signs with synchronised reference sensor signals. Sci. Data.

[4] Zhang J., Wu Y., Chen Y., Chen T. (2020). Health-radio: Towards contactless myocardial infarction detection using radio signals. IEEE Trans. Mob. Comput..

[5] Wang D., Yoo S., Cho S.H. (2020). Experimental comparison of IR-UWB radar and FMCW radar for vital signs. Sensors.

[6] Zhang Y., Yang R., Yue Y., Lim E.G., Wang Z. (2023). An Overview of Algorithms for Contactless Cardiac Feature Extraction From Radar Signals: Advances and Challenges. IEEE Trans. Instrum. Meas..

[7] Liu L., Zhang J., Qu Y., Zhang S., Xiao W. (2023). mmRH: Noncontact Vital Sign Detection With an FMCW mm-Wave Radar. IEEE Sens. J..

[8] Sun G., Tanaka Y., Kiyono K., Hashimoto K., Takase B., Liu H., Kirimoto T., Matsui T. (2019). Non-contact monitoring of heart rate variability using medical radar for the evaluation of dynamic changes in autonomic nervous activity during a head-up tilt test. J. Med. Eng. Technol..

[9] Dong S., Member G.S., Li Y., Member G.S. (2023). Accurate Detection of Doppler Cardiograms With a Parameterized Respiratory Filter Technique Using a K -Band Radar Sensor. IEEE Trans. Microw. Theory Tech..

[10] Otake Y., Kobayashi T., Hakozaki Y., Matsui T. (2021). Non-contact heart rate variability monitoring using Doppler radars located beneath bed mattress: A case report. Eur. Heart J. Case Rep..

[11] Edanami K., Kurosawa M., Yen H.T., Kanazawa T., Abe Y., Kirimoto T., Yao Y., Matsui T., Sun G. (2022). Remote sensing of vital signs by medical radar time-series signal using cardiac peak extraction and adaptive peak detection algorithm: Performance validation on healthy adults and application to neonatal monitoring at an NICU. Comput. Methods Programs Biomed..

[12] Li C., Xiao Y., Lin J. (2006). Experiment and spectral analysis of a low-power K a-band heartbeat detector measuring from four sides of a human body. IEEE Trans. Microw. Theory Tech..

[13] Xiong Y., Peng Z., Gu C., Li S., Wang D., Zhang W. (2020). Differential Enhancement Method for Robust and Accurate Heart Rate Monitoring via Microwave Vital Sign Sensing. IEEE Trans. Instrum. Meas..

[14] Mallat S.G. (1989). A theory for multiresolution signal decomposition: The wavelet representation. IEEE Trans. Pattern Anal. Mach. Intell..

[15] Huang N.E., Shen Z., Long S.R., Wu M.C., Shih H.H., Zheng Q., Yen N.C., Tung C.C., Liu H.H. (1998). The empirical mode decomposition and the Hilbert spectrum for nonlinear and non-stationary time series analysis. Proc. R. Soc. Lond. Ser. A Math. Phys. Eng. Sci..

[16] Yeh J.R., Shieh J.S., Huang N.E. (2010). Complementary Ensemble Empirical Mode Decomposition: A Novel Noise Enhanced Data Analysis Method. Adv. Data Sci. Adapt. Anal..

[17] Jang Y.I., Sim J.Y., Yang J.R., Kwon N.K. (2022). Improving heart rate variability information consistency in Doppler cardiogram using signal reconstruction system with deep learning for Contact-free heartbeat monitoring. Biomed. Signal Process. Control.

[18] Wang H., Du F., Zhu H., Zhang Z., Wang Y., Cao Q., Zhu X. (2023). HeRe: Heartbeat Signal Reconstruction for Low-Power Millimeter-Wave Radar Based on Deep Learning. IEEE Trans. Instrum. Meas..

[19] Chen J., Zhang D., Wu Z., Zhou F., Sun Q., Chen Y. (2022). Contactless Electrocardiogram Monitoring With Millimeter Wave Radar. IEEE Trans. Mob. Comput..

[20] El Abbaoui A., Sodoyer D., Elbahhar F. (2023). Contactless Heart and Respiration Rates Estimation and Classification of Driver Physiological States Using CW Radar and Temporal Neural Networks. Sensors.

[21] Pan P., Zhang Y., Deng Z., Fan S., Huang X. (2022). TFA-Net: A Deep Learning-Based Time-Frequency Analysis Tool. IEEE Trans. Neural Netw. Learn. Syst..

[22] Cw U., Radar D., Li M., Lin J. (2018). Wavelet-Transform-Based Data-Length-Variation Technique for Fast Heart Rate Detection. IEEE Trans. Microw. Theory Tech..

[23] Singh A., Rehman S.U., Yongchareon S., Chong P.H.J. (2020). Modelling of chest wall motion for cardiorespiratory activity for radar-based NCVS systems. Sensors.

[24] Nosrati M., Tavassolian N. (2018). High-Accuracy Heart Rate Variability Monitoring Using Doppler Radar Based on Gaussian Pulse Train Modeling and FTPR Algorithm. IEEE Trans. Microw. Theory Tech..

[25] Nosrati M., Tavassolian N. (2019). Accurate Doppler Radar-Based Cardiopulmonary Sensing Using Chest-Wall Acceleration. IEEE J. Electromagn. RF Microw. Med. Biol..

[26] Shafiq G., Veluvolu K.C. (2017). Multimodal chest surface motion data for respiratory and cardiovascular monitoring applications. Sci. Data.

[27] Pan J., Tompkins W.J. (1985). A Real-Time QRS Detection Algorithm. IEEE Trans. Biomed. Eng..

[28] Sedghamiz H. Matlab Implementation of Pan Tompkins ECG QRS Detector. https://www.researchgate.net/publication/313673153_Matlab_Implementation_of_Pan_Tompkins_ECG_QRS_detector?channel=doi&linkId=58a284eb92851c7fb4c1ca2d&showFulltext=true.

[29] Olshansky B., Ricci F., Fedorowski A. (2023). Importance of resting heart rate. Trends Cardiovasc. Med..

[30] Ostertagová E. (2012). Modelling using Polynomial Regression. Procedia Eng..

[31] Yang C., Tang S., Tavassolian N. (2017). Utilizing Gyroscopes Towards the Automatic Annotation of Seismocardiograms. IEEE Sens. J..

[32] Yang C., Tavassolian N. (2016). Motion Artifact Cancellation of Seismocardiographic Recording From Moving Subjects. IEEE Sens. J..

[33] Goodfellow I.J., Pouget-Abadie J., Mirza M., Xu B., Warde-Farley D., Ozair S., Courville A., Bengio Y. Generative adversarial nets. Proceedings of the 27th International Conference on Neural Information Processing Systems, NIPS’14.

[34] Hazra D., Byun Y.C. (2020). SynSigGAN: Generative Adversarial Networks for Synthetic Biomedical Signal Generation. Biology.

[35] Zhu H., Liu J., Yu J., Yang P. (2023). Artificial neural network-based predictive model for supersonic ejector in refrigeration system. Case Stud. Therm. Eng..

